# Adult Exposure to Di-N-Butyl Phthalate (DBP) Induces Persistent Effects on Testicular Cell Markers and Testosterone Biosynthesis in Mice

**DOI:** 10.3390/ijms23158718

**Published:** 2022-08-05

**Authors:** Liselott Källsten, Radwa Almamoun, Paula Pierozan, Erik Nylander, Kalliroi Sdougkou, Jonathan W. Martin, Oskar Karlsson

**Affiliations:** Science for Life Laboratory, Department of Environmental Science, Stockholm University, 11418 Stockholm, Sweden

**Keywords:** anti-androgenic, endocrine disruptor, EDC, DAZL, steroidogenesis

## Abstract

Studies indicate that phthalates are endocrine disruptors affecting reproductive health. One of the most commonly used phthalates, di-n-butyl phthalate (DBP), has been linked with adverse reproductive health outcomes in men, but the mechanisms behind these effects are still poorly understood. Here, adult male mice were orally exposed to DBP (10 or 100 mg/kg/day) for five weeks, and the testis and adrenal glands were collected one week after the last dose, to examine more persistent effects. Quantification of testosterone, androstenedione, progesterone and corticosterone concentrations by liquid chromatography-mass spectrometry showed that testicular testosterone was significantly decreased in both DBP treatment groups, whereas the other steroids were not significantly altered. Western blot analysis of testis revealed that DBP exposure increased the levels of the steroidogenic enzymes CYP11A1, HSD3β2, and CYP17A1, the oxidative stress marker nitrotyrosine, and the luteinizing hormone receptor (LHR). The analysis further demonstrated increased levels of the germ cell marker DAZL, the Sertoli cell markers vimentin and SOX9, and the Leydig cell marker SULT1E1. Overall, the present work provides more mechanistic understanding of how adult DBP exposure can induce effects on the male reproductive system by affecting several key cells and proteins important for testosterone biosynthesis and spermatogenesis, and for the first time shows that these effects persist at least one week after the last dose. It also demonstrates impairment of testosterone biosynthesis at a lower dose than previously reported.

## 1. Introduction

Phthalates are man-made chemicals that can be found in a wide array of materials and commercial products, and since the 1930s they have been the major class of chemical plasticizers [1]. Aside from their use in plastics, they are present in products such as nail polish, perfumes, drug coatings, and even skin creams [1,2]. Due to their widespread use, including as non-covalent additives in plastics, phthalates are easily emitted or leached from products and are ubiquitous in the environment, globally detectable in food, water and indoor dust [1,3,4].

Phthalates have been suggested to cause several detrimental health effects, in particular as endocrine disruptors and reproductive toxicants with anti-androgenic properties [5,6]. Di-n-butyl phthalate (DBP) has been associated with anti-androgenic effects in men [6] and is one of the most commonly detected phthalates in food [1] and human samples [6]. Epidemiological studies have demonstrated that the strongest association with phthalate exposure was semen parameters and serum testosterone levels in adult men [6]. Several in vivo toxicological studies have also shown that DBP is an endocrine disruptor, mainly by reducing testosterone levels, and that DBP exposure affects reproductive health by, for example, reducing semen motility and production [7,8]. Despite these previous studies on the effects of DBP exposure [7,9], there are still important knowledge gaps regarding the mechanisms behind these effects.

To date, most animal studies have focused on the effect of in utero or pre-pubertal exposure to DBP, even though several human studies have found correlations between DBP exposure and reproductive endpoints in adult men, such as sperm motility [6]. Studies that have examined anti-androgenic effects in rodents after adult exposure to DBP have found decreased testicular weight, sperm count and motility, decreased serum follicle-stimulating hormone and testosterone levels, as well as increased oxidative stress in the testis. However, these studies lack detailed mechanistic information about how DBP affects the testis and were based on relatively high doses [10,11,12]. Considering that all steroid hormones share the same biosynthesis pathway, it is possible that DBP can affect more than just testosterone levels. The aim of the current study was to examine persistent effects of adult male exposure to DBP on the reproductive system, with a focus on its effect on steroidogenesis and testicular cell marker expression, by collecting samples one week after the end of five weeks oral administration (10 or 100 mg/kg/day) in mice.

## 2. Results

### 2.1. General Status

All DBP-treated animals and vehicle controls survived the treatment period. The general status (e.g., skin and fur appearance, activity and responsiveness) showed no differences compared with those in the control group. No significant differences in body weight gain were observed between the groups (Supplementary Information, Table S4).

### 2.2. Steroid Hormone Concentrations in Testis and Adrenal Glands

**Figure 1:**
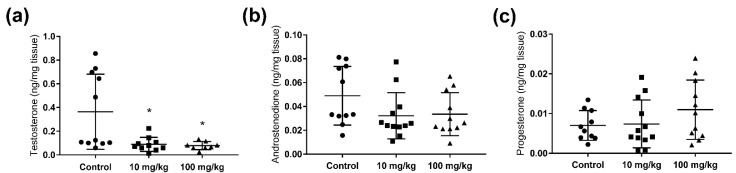
Persistent effects of five weeks oral DBP exposure on testis steroid hormone production in adult mice. Levels of testosterone (**a**), androstenedione (**b**), and progesterone (**c**) were measured by LC-HRMS in mouse testis collected one week after the last DBP administration. Values represent mean ± S.D. from 12 animals per group. Statistically significant differences from control are indicated as follows: * *p* < 0.05 (Kruskal–Wallis followed by Dunn’s test).

**Figure 2:**
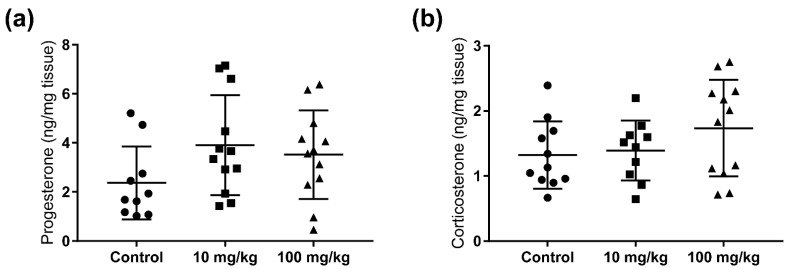
Persistent effects of five weeks oral DBP exposure on adrenal gland steroid hormone production in adult mice. Levels of corticosterone (**a**) and progesterone (**b**) were measured by LC-HRMS in mouse adrenal glands collected one week after the last DBP administration. Values represent mean ± S.D. from 12 animals per group. The statistical analysis revealed no significant group differences (Kruskal–Wallis followed by Dunn’s test).

To investigate any persistent anti-androgenic effects of five weeks adult DBP exposures in mice, the testosterone levels were measured in testicular samples collected one week after the final dose administration. Compared to controls, DBP exposure resulted in a significant decrease in testicular testosterone concentration, both in the 10 mg/kg/day (24.5%) and the 100 mg/kg/day (21.5%) dose groups (Figure 1a). To determine if DBP had a general effect on steroidogenesis, or if it specifically affected testosterone, the steroid hormones progesterone and androstenedione were also quantified in the testis extracts. No statistically significant changes were observed for these hormones. However, with increasing dose there was a decreasing trend in the level of androstenedione, and an increasing trend in the levels of progesterone in the testis (Figure 1b,c). Progesterone was also quantified in the adrenal gland, together with corticosterone, but no significant changes were observed in DBP treatment groups, although there was an increasing trend for both hormones (Figure 2).

### 2.3. Steroidogenic Protein Levels

**Figure 3:**
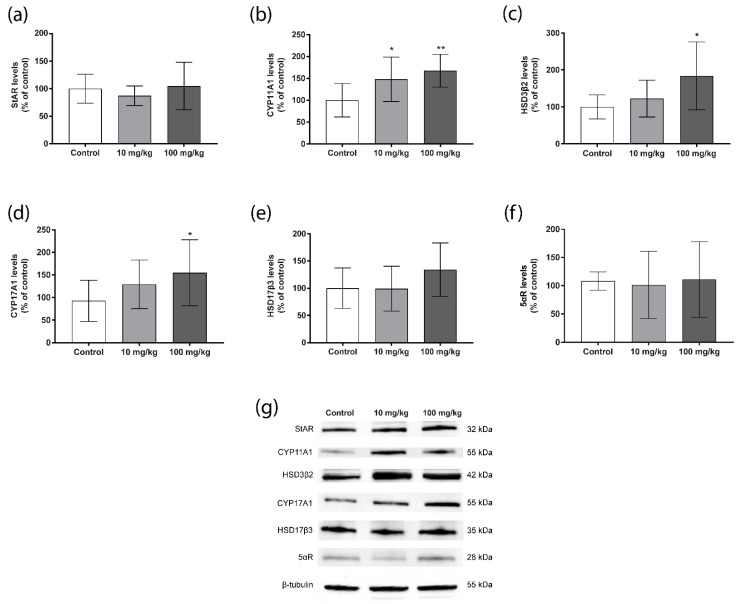
Persistent effects of five weeks oral DBP exposure on testis steroidogenic enzymes in adult mice. Levels of StAR (**a**), CYP11A1 (**b**), HSD3β2 (**c**), CYP17A1 (**d**), HSD17βB3 (**e**), and 5αR (**f**) were measured by Western blot in mouse testis collected one week after the last DBP administration. Values represent mean ± S.D. from 12 animals per group. Representative blots of three experiments are shown (**g**). β-tubulin was used as a loading control. StAR = steroidogenic acute regulator, HSD = hydroxysteroid dehydrogenase, 5αR = 5α-reductase. Statistically significant differences from control are indicated as follows: * *p* < 0.05, ** *p* < 0.01 (One-way ANOVA followed by Dunnet’s test).

To investigate the mechanisms by which DBP affects testosterone levels in mouse testis, we performed Western blot for several key steroidogenic enzymes. For the steroidogenic acute regulator (StAR), which transports cholesterol into the mitochondria, no treatment effect was detected (Figure 3a). The levels of cytochrome P450 (CYP) 11A1, which catalyses the conversion of cholesterol to pregnenolone, were significantly increased in both DBP treatment groups compared to the control (Figure 3b). Hydroxysteroid dehydrogenase (HSD) 3β2 and CYP17A1, which also catalyses early steps in steroidogenesis, showed significantly increased levels as well, but only in the 100 mg/kg/day group (Figure 3c,d). HSD17β3, which converts androstenedione to testosterone, demonstrated a similar increasing trend, although not significant (Figure 3e). The levels of 5-α reductase (5-αR), which converts testosterone into the more potent dihydrotestosterone, were not affected (Figure 3f).

**Figure 4:**
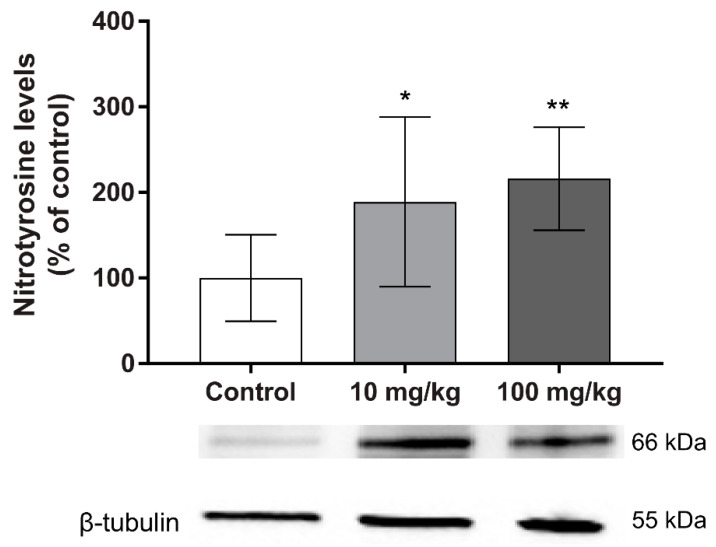
Persistent effects of five weeks oral DBP exposure on testicular oxidative stress in adult mice. Levels of nitrotyrosine were measured by Western blot in mouse testis collected one week after the last DBP administration. Values represent mean ± S.D. from 12 animals per group. Representative blots of three experiments are shown below the graph. β-tubulin was used as a loading control. Statistically significant differences from control are indicated as follows: ** *p* < 0.01 and * *p* < 0.05 (One-way ANOVA followed by Dunnet’s test).

Furthermore, we evaluated the oxidative stress protein-damage marker nitrotyrosine in testis. Interestingly, a significant and dose-dependent increase was observed in the groups treated with DBP, compared with the control group (Figure 4).

**Figure 5:**
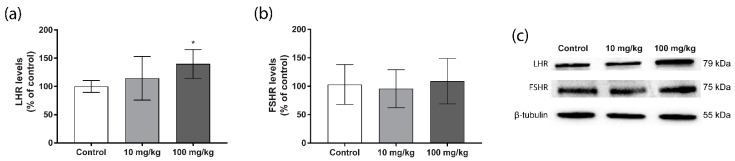
Persistent effects of five weeks oral DBP exposure on hormone receptors in adult mice. Levels of luteinizing hormone receptor (LHR; (**a**)) and follicle stimulating hormone receptor (FSHR; (**b**)) were measured by Western blot in mouse testis collected one week after the last DBP administration. Values represent mean ± S.D. from 12 animals per group. Representative blots of three experiments are shown (**c**). β-tubulin was used as a loading control. Statistically significant differences from control are indicated as follows: * *p* < 0.05 (One-way ANOVA followed by Dunnet’s test).

To further explore the mechanisms by which DBP-altered testosterone levels in the testis, we measured the levels of the luteinizing hormone receptor (LHR) and follicle-stimulating hormone receptor (FSHR). The results revealed a dose-dependent increase in the levels of LHR, which was statistically significant in the 100 mg/kg/day DBP group (Figure 5a). No alteration in the levels of FSHR were observed (Figure 5b).

### 2.4. Testis Histology and Cell Marker Levels

**Figure 6:**
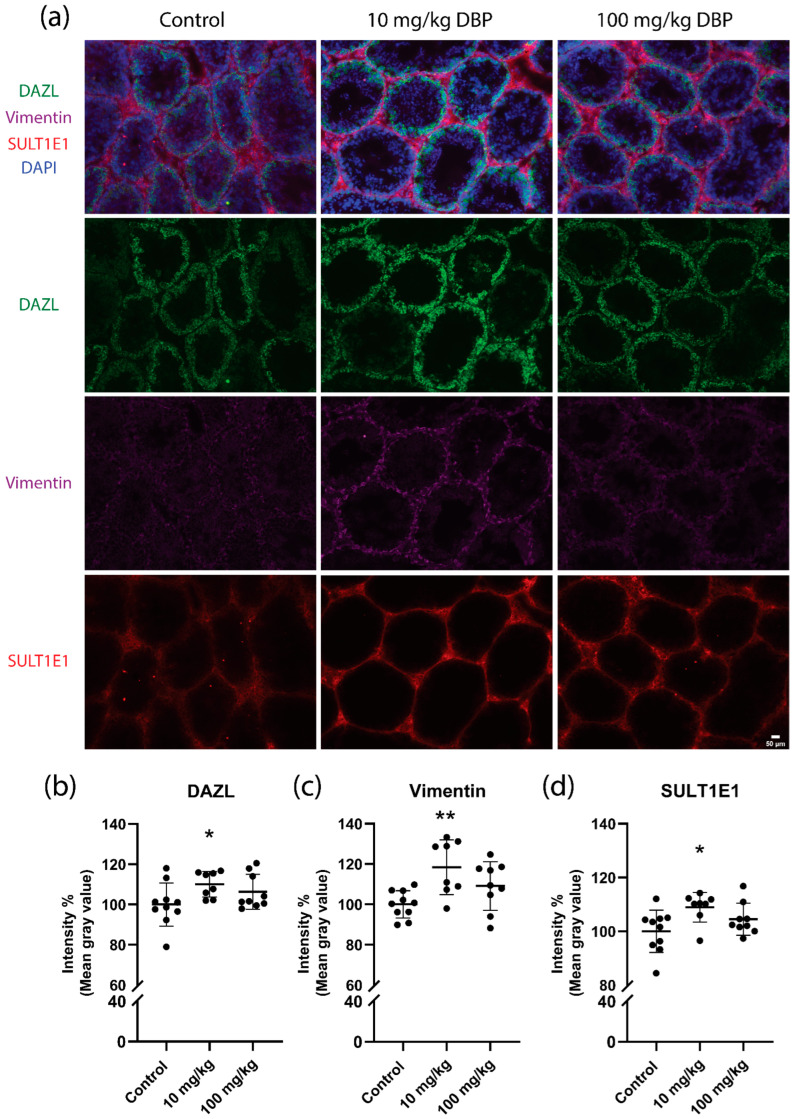
Persistent effects of five weeks oral DBP exposure on testicular cell markers in adult mice. Representative images of testis cross-sections from DBP-treated mice or control (**a**). DAZL (green), vimentin (purple) and SULT1E1 (red) by immunofluorescence in cryosections from mouse testis collected one week after the last DBP administration. The nuclear marker DAPI is shown in blue. Fluorescence intensity quantification of DAZL, vimentin and SULT1E1 is shown as a percentage of control (**b**–**d**). Data are represented as mean ± SD (*n* = 8–11 animals per group). Statistically significant differences from control are indicated as follows: * *p* < 0.05 and ** *p* < 0.01 (Kruskal–Wallis test followed by Dunn’s test). Scale bar: 50 µm.

We next examined the expression of three key testicular proteins by immunofluorescence in the testis: RNA binding protein DAZL (germ cells), vimentin intermediate filaments (Sertoli cells), and the sulfation conjugating enzyme SULT1E1 (Leydig cells). Representative images are shown in Figure 6a. Immunofluorescence analysis of cryo-sections revealed a small yet statistically significant increase in staining intensity of DAZL (11%), vimentin (19%) and SULT1E1 (10%) in 10 mg/kg/day DBP-treated mice relative to controls (Figure 6b–d). A similar trend was seen in the 100 mg/kg/day DBP group, but the increase did not reach statistical significance.

**Figure 7:**
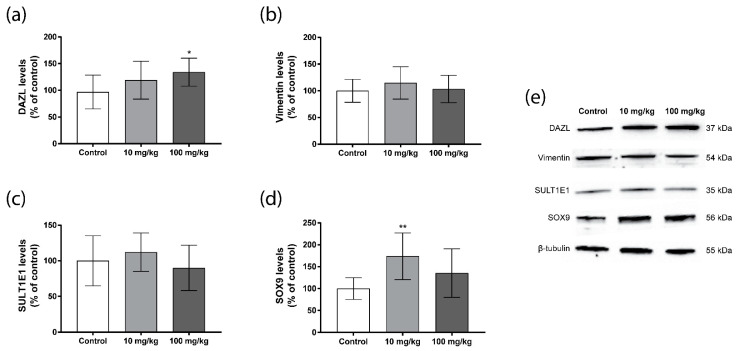
Persistent effects of five weeks oral DBP exposure on testicular cell markers in adult mice. Levels of DAZL (**a**), vimentin (**b**), SULT1E1 (**c**), and SOX9 (**d**) were measured by Western blot in mouse testis collected one week after the last DBP administration. Values represent mean ± S.D. from 12 animals per group. Representative blots of three experiments are shown (**e**). β-tubulin was used as a loading control. Statistically significant differences from control are indicated as follows: ** *p* < 0.01 and * *p* < 0.05 (One-way ANOVA followed by Dunnet’s test).

The Western blot analysis of these cell markers showed similar results as the immunofluorescence analysis of the cryosections, with some slight differences. The results for the germ cell marker DAZL showed a dose-dependent increase, which was significant for the 100 mg/kg/day group compared with the controls (Figure 7a). For vimentin and SULT1E1, a small non-significant increase was observed in the 10 mg/kg/day group (Figure 7b,c). Moreover, a significant increase in SOX9, another Sertoli cell marker, was observed in the 10 mg/kg/day group (Figure 7d).

Assessment of the diameter and area of the seminiferous tubules demonstrated a non-significant trend towards an increase in diameter and area of the tubules in the DBP-treated groups (Supplementary information, Figure S4).

## 3. Discussion

Several studies have demonstrated that DBP can be an endocrine disruptor of the reproductive system [5,6,7]. However, to our knowledge only six of these former studies focused on effects on the reproductive system after adult male exposure in mammals [8,10,11,12,13,14]. Here, we investigated the effects on the reproductive system one week after the end of five weeks oral exposure to DBP (10 or 100 mg/kg/day) in adult male mice, to examine more persistent impairments. Interestingly, the results demonstrated that DBP reduced the testicular testosterone levels, increased steroidogenic enzyme levels and LHR levels, induced oxidative stress and affected the testis cell markers DAZL, vimentin, SOX9 and SULT1E1.

DBP exposure induced reduced testosterone levels, in parallel with an increase in the testicular levels of several steroidogenic enzymes, which would be expected to increase the hormone synthesis. This indicates that DBP also affects enzyme function, which could lead to the reduced testosterone levels as well as a compensatory upregulation of the enzyme levels. Since oxidative stress can affect the enzyme function through protein oxidation [15,16,17], we analysed the levels of nitrotyrosine, a marker of protein damage by oxidative stress. We observed increased levels in both treatment groups, suggesting that a disruption of enzyme function could be due to reactive species production. This is in accordance with several studies showing that phthalates elicit oxidative stress [10,12] and disrupt steroidogenesis [9,10,18]. However, more studies are necessary to fully elucidate how DBP exposure is affecting the steroidogenic enzymes. The observed increase in LHR could also be a result of positive feedback in the hypothalamic–pituitary–testicular (HPT) axis due to the decreased testosterone levels [19]. Hypothalamic gonadotropin-releasing hormone (GnRH) is the central regulator of the male reproductive hormonal cascade, via the HPT axis. GnRH stimulates the release of LH, which stimulates Leydig cells to produce testosterone [20].

Out of the four steroid hormones analysed in this study, only testosterone was significantly changed. The fact that the levels of androstenedione, the immediate precursor of testosterone, only had a slight and non-significant tendency towards a decrease suggests that DBP specifically affects the testosterone production. There are only a few studies that have found changes in the levels of other steroid hormones after phthalate exposure. These are mostly in vitro studies and with other phthalates than DBP, making it difficult to compare with our results. One study with rats exposed to 500 mg/kg/day DBP in utero (GD 12–21) observed a significant decrease in both testosterone and androstenedione levels, while progesterone was affected differently depending on the days of exposure [21]. This supports our finding that DBP, in particular, affects the production of androgens. On the other hand, a recent study found that 6–8week-old rats exposed to 100–1000 mg/kg/day DBP for two weeks had significantly decreased corticosterone levels at doses of 500 mg/kg or higher [22], which is in contrast with our results that demonstrated a slight but non-significant increase in corticosterone concentration. However, since no significant effect was observed at the 100 mg/kg/day dose, it is possible that the mechanism differs depending on the dose. It should be noted that their study did not measure the effects on any other steroid hormones, and the effect on corticosterone may also be more short-lived, as they measured the hormone levels 24 h after the last dose [22], whereas the samples in the current study were collected one week after the final dose to allow examination of more persistent effects.

In addition to the steroidogenic perturbations, adult DBP exposure increased the levels of the key testicular proteins and cell markers DAZL, vimentin and SULT1E1, one week after the end of five weeks oral administration in male mice. These proteins are expressed by the three testicular cell populations: germ cells, Sertoli, and Leydig cells, respectively [23,24,25]. DAZL is a germ cell-specific RNA binding protein, expressed by spermatogonia and spermatocytes [24], which is essential for promoting spermatogenesis by regulating the transcription of necessary germline genes [26,27]. Loss of DAZL expression in mice results in germ cell apoptosis and disruption of meiosis, indicating that DAZL is critical for germ cell survival [24,27,28], whereas in vitro, DAZL overexpressed cells exhibited altered morphology with spermatogonia-like colonies and affected the transcription of several genes involved in germ cell differentiation and germ cycle arrest [29]. DAZL binds to the 3’ untranslated regions and promotes translation of genes, which include regulators of transcription and RNA metabolism [30]. The DBP-induced DAZL upregulation will therefore likely affect the spermatogenesis, as well as the sperm transcriptome and proteome. This finding can be related with previous studies showing that DBP decreased spermatozoa mobility, sperm count, caused degeneration of seminiferous tubules and increased abnormal spermatozoa in rodents at several experimental designs [7].

The consequences of the DBP-induced increase in testicular vimentin levels may also be severe. Vimentin is a mesenchymal cell intermediate filaments protein with diverse functions [31]. In Sertoli cells, vimentin has a critical role in spermatogenesis and maintaining the structural integrity of the seminiferous epithelium [23,32]. Consistent with our results, it has been shown in vitro that exposure to the DBP metabolite monobutyl phthalate (MBP) upregulated vimentin expression in the murine Y1 and MLTC-1 tumour cell lines [33]. The increased vimentin expression resulted from activation of the NF-kB pathway and demethylation of the vimentin promoter [33]. In rats, it has been shown that in utero (GD 12–20) exposure to 500 mg/kg/day DBP-induced transient effects on vimentin cytoskeleton organization indicating abnormal contact between Sertoli and germ cells in fetal life [34]. In our animal model DBP exposure also increased the levels of SOX9, another marker of Sertoli cells which is expressed during all maturation stages [35]. SOX9 is a DNA-binding transcription factor that, in addition to its crucial role in testis development [36], controls adult testis maintenance [37]. Overexpression of SOX9 has been shown to result in testicular abnormalities in mice [38].

The third affected testicular key protein, SULT1E1, is an enzyme central for estrogen regulation and homeostasis in the testis. It catalyzes the sulfate conjugation of estrone and estradiol, leading to their inactivation and inability to bind to the estrogen receptors in the testis [39,40]. Expression of SULT1E1 appears to be a unique feature of the adult type of Leydig cells and is under control of LH [41,42]. The observed increase in SULT1E1 in the DBP-exposed mice could therefore be the result of increased levels of LHR, and that may affect estrogen signalling in the testis.

The decrease in testicular testosterone observed in our animal model is in line with previous studies [10,21,43]. However, our results demonstrate a significant testosterone decrease already in the 10 mg/kg/day group, which was the lowest dose tested, and a much lower dose than most other studies have included [7]. For instance, in the study by Aly and colleagues, they also detected a significant decrease in testosterone after adult mice were exposed to DBP for 15 days, but their lowest dose was 200 mg/kg/day [10]. An in utero (GD 12–19) exposure study in male rats included DBP doses as low as 0.1 mg/kg/day, but only observed significant decrease in foetal testicular testosterone at 50 mg/kg/day or higher [44]. This difference could be due to species differences, shorter exposure time, or that the actual dose which reached the foetus was lower than the maternal dose, it may also indicate that adult testosterone biosynthesis is more sensitive to DBP exposure.

The DBP levels that humans are exposed to vary greatly between individuals, with one study from Germany reporting an estimate of median daily intake of DBP at 2.1 µg/kg. The subject with the highest levels in the study had an estimated intake of 230 µg/kg [45]. Even more extreme DBP exposures have been detected in patients taking medicine with DBP in their coating, such as Asacol, a medicine against irritable bowel syndrome, where patients have been found to have levels of DBP metabolites in their urine which are >500 times higher than the average concentrations found in the general US population [46]. The doses used in the current study are higher than the average human exposure, and even the lowest dose is probably a few times higher than the extreme human exposure, also considering metabolic differences and allometric scaling [47]. However, it should be noted that this study examined the effect of DBP exposure for five weeks, followed by a one-week non-exposure period, whereas humans are chronically exposed to phthalates as a result of the ubiquitous use of phthalates in personal-care and consumer products [48].

Once ingested, DBP is rapidly and extensively (>80%) metabolised to MBP by esterases in the intestine [49,50], and to a smaller extent further metabolised by side-chain oxidation or glucuronidation [51,52]. In both humans and rodents the elimination half-life of DBP is short, and >70% of a given dose is excreted within 24 h, mainly in the form of MBP [51,53,54]. Considering this rapid elimination of the compound, it is interesting that significant alterations can be detected even one week after the last dose. Persistent effects of DBP exposure on steroidogenesis have previously been observed after in utero exposure to 100–500 mg/kg/day DBP on GD 1, 7 and 14, where decreased testosterone was observed in the prenatally exposed rats when they were 100 days old [55], but our study is a first indication that adult exposure might also induce more persistent effects on testosterone biosynthesis and key testicular proteins. In contrast to studies reporting that in utero exposure to ≥50 mg/kg/day DBP resulted in abnormalities in shape and dilation of the seminiferous tubules [56], we only observed a trend towards an increase in the diameter and area of the tubules with DBP treatment. This discrepancy indicates that adult exposure to DBP might have less impact on tubules morphology than foetal exposure.

Our results support that the adult testis is a central target organ for the ubiquitous contaminant DBP, and revealed significant anti-androgenic effects even at the lowest dose tested (10 mg/kg/day). To our knowledge, the lowest observed adverse effect level (LOAEL) from our animal model is lower than previously reported no observed adverse effect level (NOAEL) for impaired testosterone biosynthesis. The study also shows for the first time that several of the effects persist at least one week after the end of the exposure. Our findings provide mechanistic understanding of how adult male exposure to DBP can induce these persistent effects in the reproductive system, by affecting several key testicular cells, and proteins important for the testosterone biosynthesis pathway and spermatogenesis. One week after the last DBP dose was administered, we found decreased testosterone levels, altered levels of several steroidogenic enzymes, increased LHR levels, induced oxidative stress, and increased DAZL, vimentin, SOX9 and SULT1E1 levels in testis. This can have important implications for male reproductive health. Testosterone and vimentin are for example vital for the spermatogenesis, and the germ cell upregulation of DAZL may affect both sperm development and sperm biomolecule levels as it is central for the spermatogenesis and promotes translation of genes, including regulators of transcription and RNA metabolism.

## 4. Methods and Materials

### 4.1. Chemicals and Reagents

DBP (CAS No 84-74-2, purity > 99%), corn oil (CAS No 8001-30-7), analytical grade standards of testosterone, androstenedione, corticosterone and progesterone, isotope-labelled testosterone-d3, corticosterone-d4 and progesterone-^13^C5, SIGMAFAST™ protease inhibitor tablets, DAPI, bovine serum albumin (BSA), and DPX-mounting medium were all purchased from Sigma Aldrich (St. Louis, MO, USA). Tween 20, NaCl and Tris-HCl, as well as LC-MS grade water, methanol and acetonitrile were all purchased from Fisher (Loughborough, UK). Ammonium fluoride was purchased from Honeywell Research Chemicals (Morris Plains, NJ, USA). HPLC grade ethyl acetate was purchased from Rathburn Chemicals Ltd. (Walkerburn, UK). Phosphate buffered saline (PBS) was purchased from Gibco, Life Technologies Europe (Bleiswijk, The Netherlands). Triton X-100 and analytical grade methanol (used for immunohistochemistry) were purchased from VWR international (Radnor, PA, USA). Normal goat serum, F(ab’)2 goat anti-mouse immunoglobulin G was purchased from Invitrogen (Waltham, MA, USA). TBS was purchased from Medicago AB (Uppsala, Sweden). Sodium deoxycholate was purchased from Alfa Aesar ThermoFisher GmbH (Kandel, Germany). Dry milk was purchased from Cell signalling Technology (Danvers, MA, USA). The chemiluminescence ECL kit was purchased from Biorad (Hercules, CA, USA).

### 4.2. Animal Housing and Exposure

Adult male C57bl/6N mice were purchased from Charles River (Sulzfeld, Germany) and housed in Makrolon cages in a room controlled for temperature and humidity. The light cycle was set as 12 h light/12 h dark. Drinking water and food pellets were provided ad libitum. The animals were housed four individuals per cage, four cages per group, and were acclimated for one week before the beginning of the treatment. At eight-weeks-old, the mice were treated via gavage once a day for five weeks (*n* = 12 per treatment group). Gavage solutions contained either 1 mg DBP/mL corn oil (i.e., for dose of 10 mg/kg/day) or 10 mg DBP/mL corn oil (i.e., for dose of 100 mg/kg/day), or corn oil only (vehicle control group). To study persistent effects on the male reproductive system, the mice were euthanized by decapitation seven days after the last dose was administrated, and the testes and adrenal glands were collected and stored at −80 °C until analysis. 

### 4.3. Steroid Hormone Extraction

Approximately half (46–95 mg) of one testis from each animal was used for the extraction of steroids. Each part was weighed and placed in a plastic tube together with a 5 mm stainless steel bead and methanol (10 times the tissue weight in volume, i.e., 1 mg tissue = 10 µL methanol). The tissue was homogenised with a TissueLyser II (Qiagen, Hilden, Germany) for 1.5 min at 25 Hz. The homogenate was then stirred for 10 min on a nutating mixer (VWR International, Radnor, PA, USA). After removing the bead, the samples were centrifuged for 10 min at 13,000× *g* and the supernatant was collected. Further purification of the tissue extract was done by mixing 50 µL of the supernatant with 250 µL acetonitrile:ethyl acetate (9:1) with internal standards (IS) added (Supplementary information). The mixture was stirred for 12 min to precipitate any remaining proteins, and then centrifuged at 14,500× *g* for 10 min. The supernatant was carefully transferred to clean tubes and evaporated until complete dryness under a stream of nitrogen gas in a TurboVap (Biotage, Uppsala, Sweden). The dried samples were then reconstituted in 50 µL methanol:water (30:70) and filtered with 0.2 µm nylon centrifuge filters before analysis. Quality control (QC) samples were prepared by pooling 10 µL of supernatant from the first centrifugation step from all samples, and then followed the same procedure as the other samples. Calibration standards for seven different calibration points were prepared in solvent (methanol:water, 30:70) with IS added at the same concentrations as in the samples (Supplementary information, Table S1).

The method above was also used for adrenal glands, with the exception that both adrenal glands from each animal were pooled together for homogenisation, the volume of methanol for the homogenisation was 100 times the tissue weight, and after evaporation the reconstitution volume was 250 µL, i.e., a 5-fold dilution.

The recovery and precision of the extraction method was validated before the samples were analysed (Supplementary information, Section 1.3, Table S2). 

### 4.4. UPLC-HRMS Analysis of Steroid Hormone Levels

Chromatographic separation was achieved using an ultra-high pressure liquid chromatography (LC) system (Ultimate 3000, Thermo Scientific, Sunnyvale, CA, USA) with an Acquity UPLC BEH C18 analytical column (2.1 × 50 mm, 1.7 µm particles) and VanGuard Acquity UPLC BEH C18 pre-column (2.1 × 5 mm, 1.7 µm particles) (Waters, Milford, MA, USA). The mobile phases consisted of water with 1 mM ammonium fluoride (A) and methanol (B). The flow rate was set as 0.3 mL/min and the column temperature was 50 °C. Mass spectral data were acquired with a Q-Exactive HF-X Orbitrap (Thermo Fisher Scientific, Bremen, Germany) high-resolution mass spectrometer (HRMS), employing electrospray ionisation in positive mode. Data was acquired in full scan mode (MS1) and parallel reaction monitoring (PRM; MS2). More details of the LC-HRMS method can be found in the supplementary information (Section 1, Figures S1–S3).

### 4.5. Immunohistochemistry

Cryosections of 12 µm were prepared from mouse testis using a cryostat (CryoStar NX70, Thermo Scientific, Sunnyvale, CA, USA). The sections were fixed with ice-cold methanol for 5 min and washed three times with PBS. After fixation, the sections were blocked for 3 h in blocking solution (10% normal goat serum) with 1% F(ab’)2 goat anti-mouse immunoglobulin G diluted in IF buffer (composed of 0.2% Triton X-100, 0.05% Tween 20, and 0.1% BSA diluted in PBS). The slides were then simultaneously incubated with antibodies against DAZL, vimentin and SULT1E1 (Supplementary information, Table S3), diluted in IF buffer, overnight at 4 °C. The sections were washed three times in IF buffer, followed by 45 min incubation at room temperature with the following secondary antibodies: goat anti-mouse Alexa Fluor 555 (1:500, Invitrogen A32727), goat anti-rabbit Alexa Fluor 488 (1:500, Invitrogen A11034) and goat anti-chicken Alexa Fluor 647 (1:500, Invitrogen A21449), diluted in IF buffer. The slides were then washed once with IF buffer and twice with PBS. The nuclei were counterstained with DAPI (0.5 µg/mL) for 5 min at room temperature and washed with PBS for 10 min before the sections were mounted using DPX-mounting media. Negative controls that omit the primary antibody were performed. Images were captured with a fluorescent microscope (Olympus BX53F2, Tokyo, Japan) using 20× magnification and constant acquisition settings for all the samples. To quantify fluorescence signal intensity, the mean gray value was measured (as a percentage of control) using the ImageJ software (NIH, USA). The morphological analysis of the seminiferous tubules was performed using semi-automated image analysis in ImageJ. Briefly, stacks of acquired images were manually thresholded and converted to binary images. The tubules were then detected using the “analyse particles” function and selections were visually checked prior to continuation of the automated analysis. Both the area and the Feret’s diameter of the tubules were calculated and summarized.

### 4.6. Western Blot

Sections of one testis per animal were lysed with RIPA buffer (150 mM NaCl, 0.1% triton X-100, 0.5% sodium deoxycholate, 0.1% SDS, 50 mM Tris-HCl, 1% protease inhibitors pH 8). The protein content was measured by the Lowry assay [57] and 20 µg of protein per sample was separated by SDS-page and Western blot was performed as previously described [58]. The primary antibodies (Supplementary information, Table S3) were diluted in TTBS with 5% of dry milk and incubated overnight. After that, the blots were incubated for 1 h in TBS containing peroxidase conjugated mouse anti-rabbit or mouse IgG diluted 1:5000 (Abcam, ab7090 and ab47827, respectively). The blot was developed with a chemiluminescence ECL kit using a charge-coupled device (CCD) imager (iBright CL750 Imaging System; ThermoFisher, Rockford, IL, USA), and the optical density was measured with ImageJ. The loading control β-tubulin (Abcam, ab6046) was used for normalization. The protein levels are shown as percentage of the control.

### 4.7. Statistical Analysis

Results are presented as mean ± standard deviation (S.D.) for each group. Differences compared to the control were analyzed by one-way analysis of variance (ANOVA) followed by Dunnet’s multiple comparison test. For data that did not pass the Shapiro–Wilk normality test, differences compared to control were examined with the Kruskal–Wallis test instead, followed by Dunn’s multiple test correction. Outliers were identified and excluded using the ROUT method [59], with Q = 1%. All statistical tests were done using Prism 7 (GraphPad Software, San Diego, CA, USA).

## Data Availability

The data presented in this study are available on request.

## Supplementary Materials

The Following supporting information can be downloaded at: https://www.mdpi.com/article/10.3390/ijms23158718/s1.
