# Supplementary material for: Adult Exposure to Di-N-Butyl Phthalate (DBP) Induces Persistent Effects on Testicular Cell Markers and Testosterone Biosynthesis in Mice
Source: Int J Mol Sci. 2022 Aug 5;23(15):8718. doi: 10.3390/ijms23158718 (PMC9369267; doi:10.3390/ijms23158718)
Supplement: Supplementary file 1 [file ijms-23-08718-s001.zip › ijms-1803299-supplementary.pdf]

Article

Adult Exposure to Di-N-Butyl Phthalate (DBP) Induces Persistent Effects on Testicular Cell Markers and Testosterone Biosynthesis in Mice

Liselott Källsten †, Radwa Almamoun †, Paula Pierozan, Erik Nylander, Kalliroi Sdougkou, Jonathan W. Martin and Oskar Karlsson *

Science for Life Laboratory, Department of Environmental Science, Stockholm University, 11418 Stockholm, Sweden
* Correspondence: oskar.karlsson@aces.su.se
† These authors contributed equally to this work.

1. MATERIAL AND METHODS

1.1 Calibration curve

Since no blank matrix was available, calibration standards were prepared in solvent.
Standard stock solutions of the steroids were prepared in methanol at concentrations of 2 mg/ml. These were then diluted and mixed to generate calibration standards (Table S1). A mix of isotope labelled internal standards (testosterone-d3, corticosterone-d4 and progesterone-^{13}C5) was added to each calibration sample to a final concentration of 5 ng/ml.
All calibration points were prepared in triplicate from stock solutions on the day of analysis. The final dilution consisted of 25% methanol stock solution and 75% water.

1.2 LC-HRMS method

1.2.1 LC gradient

A linear gradient run was used for chromatographic separation of the hormones. The mobile phases were as follows: A = LC-MS grade water with 0.1 % ammonium fluoride, B = 100% LC-MS grade methanol. The following linear gradient was used: 30% B from 0-0.5 min, 30% - 100% B from 0.5 to 6.5 min, hold at 100% B from 6.5. to 9.5 min and then back to starting conditions for 2.5 min. Total run time 12.5 min.

1.2.2 HRMS method

All samples were analysed with electrospray ionisation in positive full scan mode (scan range 80-450 m/z), with and additional parallel reaction mode (PRM) in the same run. Full scan was used for quantification and PRM was only used to confirm the identity of the compounds. This was done by matching the fragmentation spectrum (Figures S1-S3) and retention time of the analyte in the sample with that of a pure standard. For PRM, an inclusion list of the target analytes $[M+H]^+$, including the isotope labelled standards, was defined with 30 s windows set for each analyte included. The resolution of the full scan mode was set at 60 000 (FWHM at 200 m/z), and for PRM it was set at 30 000. The settings of the mass spectrometer were: capillary temperature 300°C, spray voltage 4 kV and funnel RF level 40. Fragmentation was performed at collision energy 30 eV.

1.3. Validation

1.3.1 LOQ and linearity

The lower limit of quantification (LLOQ) was defined as the lowest concentration in the calibration curve with a coefficient of variation (CV%) <20 %, a signal to noise ratio (S/N) above 10, and a peak with a minimum of 10 scans. With this definition, the LLOQ was determined to be 0.025 ng/ml for testosterone, progesterone and androstenedione, and 0.25 ng/ml for corticosterone. The linearity of the calibration curve was high for all analytes, with R^2 >0.99, and the CV% was below 10% for all calibration points, except for the lowest concentration of androstenedione that was 29%.

1.3.2 Recovery

Recovery of the extraction method was determined by spiking pooled tissue homogenate with 10 ng/ml, and comparing this with pooled samples spiked after the extraction. Due to high concentration of testosterone and androstenedione in the testis homogenate, the recovery of these were determined in adrenal gland homogenate, and vice versa for corticosterone and progesterone in adrenal gland. Recovery of the method was calculated with the formula %

Recovery = ((peak area of pre-extraction-spiked tissue sample – non-spiked tissue sample) / (peak area of post-extraction-spiked tissue sample – non-spiked tissue sample)) × 100.
The recovery of the hormones varied between 76 and 94% (Table S2).

1.3.3 Precision
Precision of the analytical method was calculated in terms of coefficient of variance (CV%) for the replicate analysis of quality control samples that should be ≤20% as the acceptance criterion. QC samples (consisting of pooled tissue homogenate) were extracted in triplicate and analysed. The CV% varied between 1.2% and 3.9% for the different analytes (Table S2).

2. SUPPLEMENTARY TABLES

Table S1. Concentrations of the analytes in the calibration curve.

Calibration point	Testosterone	Androstenedione	Corticosterone	Progesterone
	(ng/ml)			
1	0.025	0.0125	0.25	0.025
2	0.05	0.025	0.5	0.05
3	0.25	0.125	2.5	0.25
4	0.5	0.25	5	0.5
5	2.5	1.25	25	2.5
6	10	5	100	10
7	50	25	500	50

Table S2. Recovery and precision data for the quantified steroid hormones.

Analyte	Recovery (%)	Precision (CV %)
Testosterone	76	2.2
Androstenedione	78	3.9
Corticosterone	83	2.4
Progesterone	84	1.2

Table S3. Primary antibodies used for Western blot (WB) and immunohistochemical (IH) analysis of mouse testis.

**Antibody**	**Dilution**	**Cat. no./ Manufacturer**
DAZL	1:5000 (WB)<br>1:1000 (IH)	ab34139 Abcam (Cambridge, UK)
Vimentin	1:5000 (WB)<br>1:1000 (IH)	ab24525 Abcam
SULT1E1	1:3000 (WB)<br>1:200 (IH)	sc-376009 Santa Cruz (Dallas, TX, USA)
SOX9	1:5000	Ab185966 Abcam
StAR	1:5000	Ab 133657 Abcam
CYP11A1	1:1000	ab175408 Abcam
CYP17A1	1:5000	ab125022 Abcam
HSD3β2	1:2000	PA5-27791 Invitrogen (Waltham, MA, USA)
HSD17β3	1:2000	MBS8306928 MyBioSource (San Diego, CA, USA)
LHR	1:2000	PA5-97923 Invitrogen
Nitrotyrosine	1:1000	05-233 Sigma-Aldrich (St Louis, MO, USA)
5α-reductase	1:2000	MA5-37985 Invitrogen
FSHR	1:2000	PA5-50963 Invitrogen

Table S4. Body weights of the mice before and after treatment.

	**Control**	**10 mg/kg**	**100 mg/kg**
	g (± S.D.)		
Weight before treatment	21.03 ± 0.69	21.31 ± 1.42	21.29 ± 0.78
Weight after treatment	23 ± 0.75	23.59 ± 1.91	23.38 ± 1.08

3. SUPPLEMENTARY FIGURES

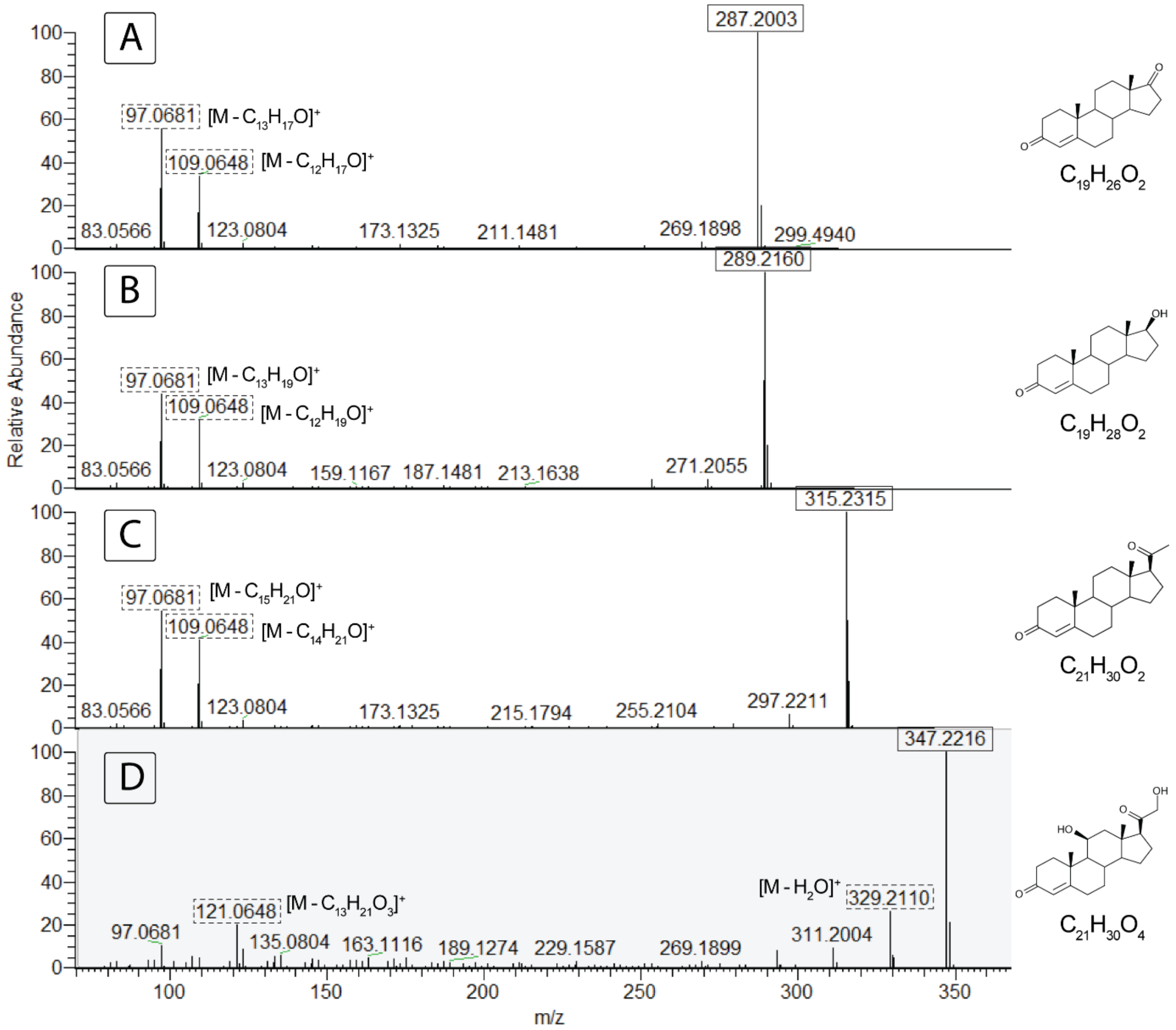

Figure S1. PRM fragmentation spectra for standard solutions (0.5 ng/ml) of androstenedione (A), testosterone (B), progesterone (C), and corticosterone (D). The parent ion $[M+H]^+$ for each compound has been marked with a solid border, and the main fragment ions are marked with dashed borders and annotated with their respective formulas. The chemical structure and formula of the parent compounds are inserted to the right of their respective spectrum. Collision energy = 30 eV.

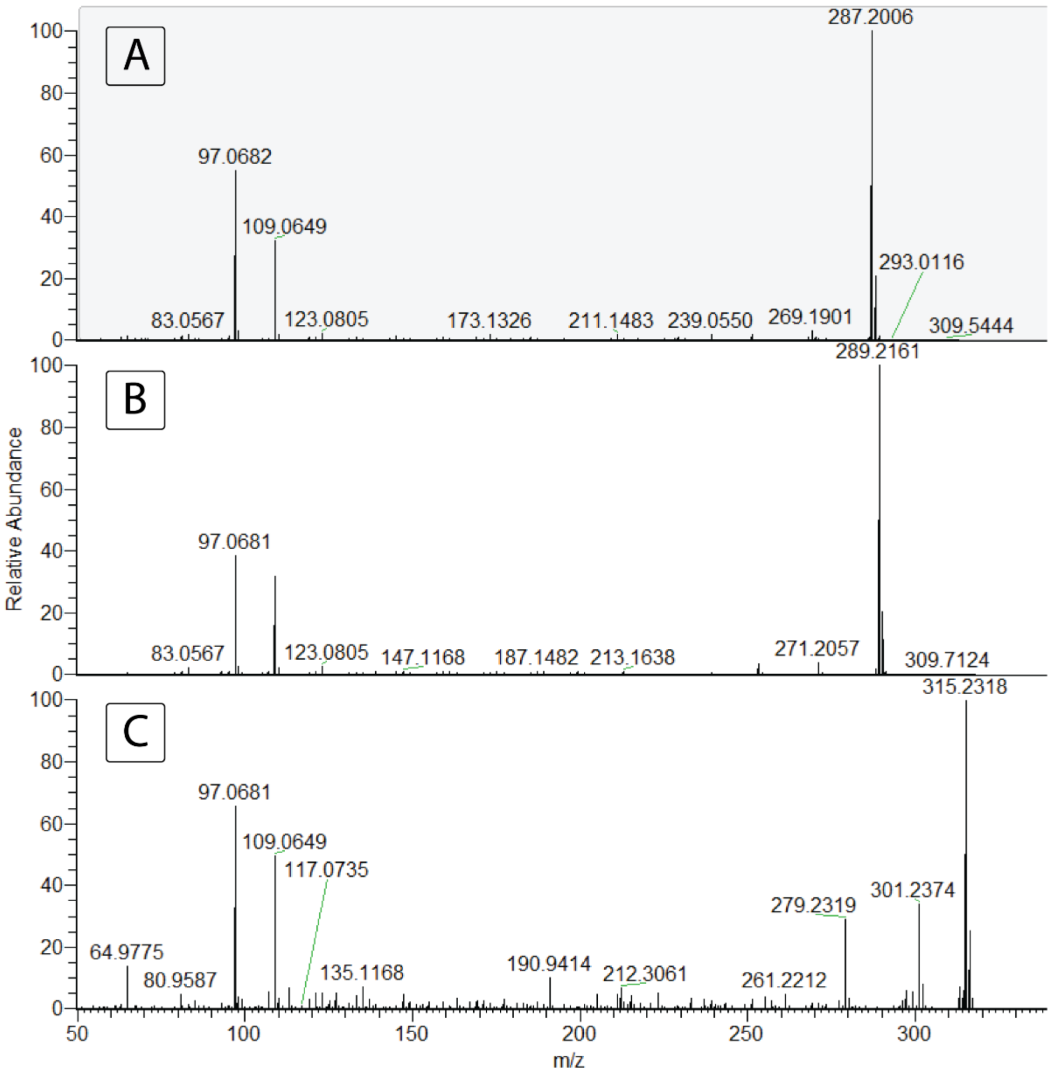

Figure S2. PRM fragmentation spectra for androstenedione (A), testosterone (B), and progesterone (C) in a testis sample. Collision energy = 30 eV.

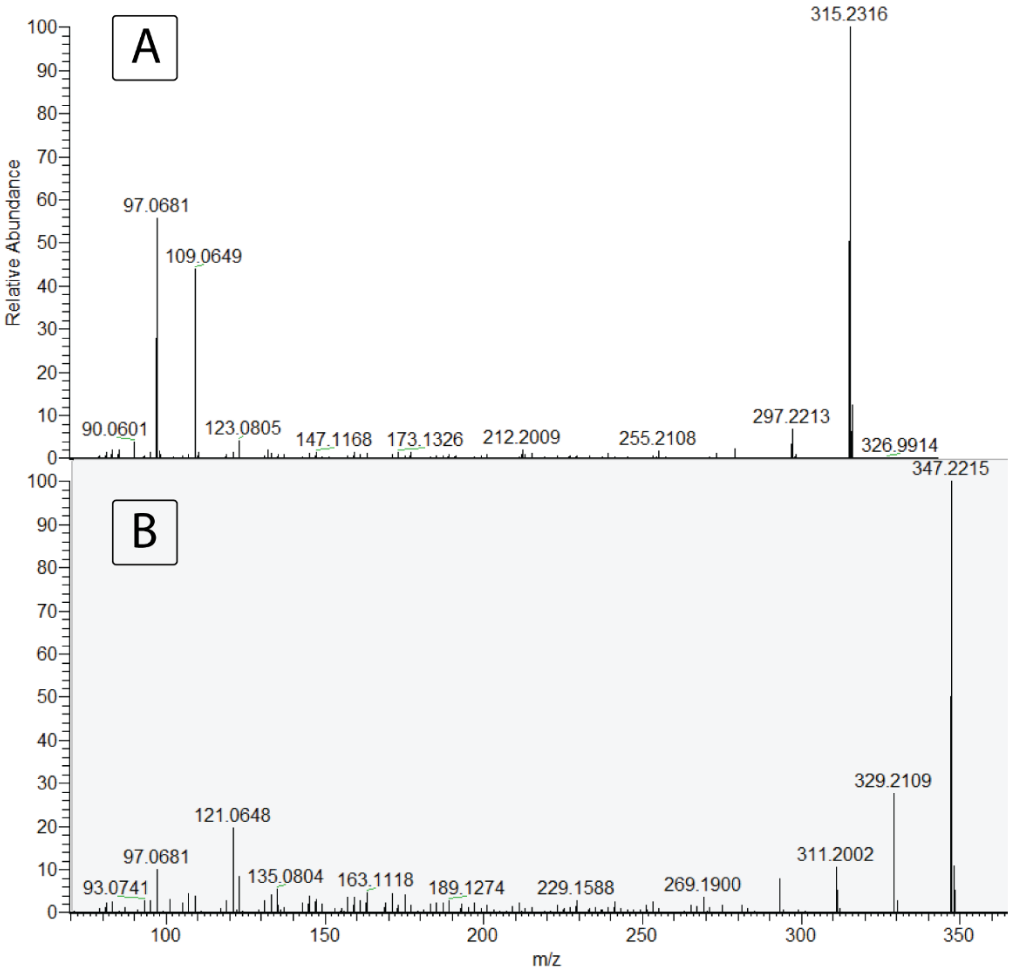

Figure S3. PRM fragmentation spectra for progesterone (A) and corticosterone (B) in an adrenal gland sample. Collision energy = 30 eV.

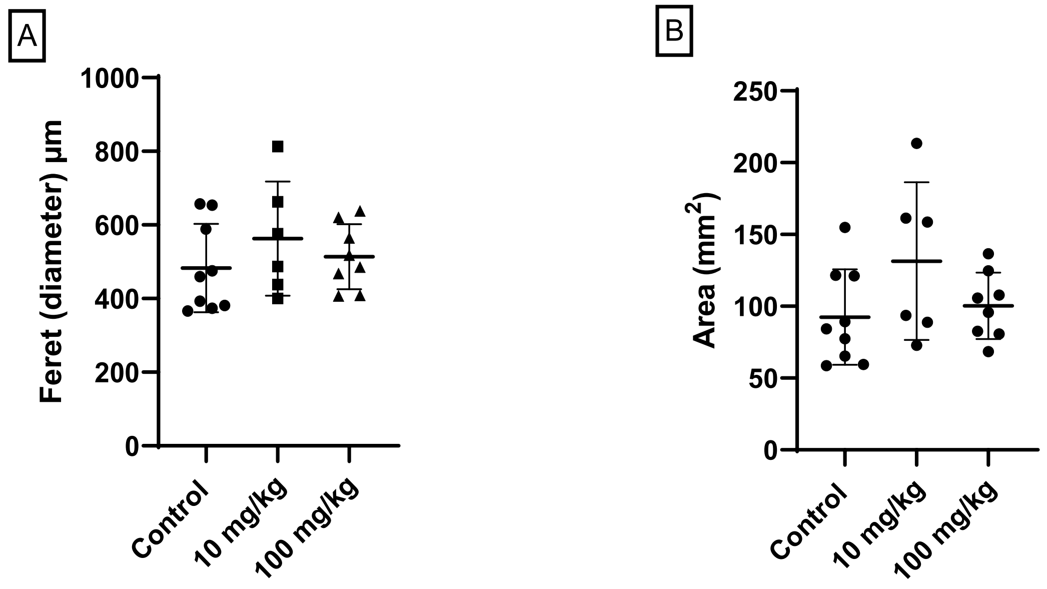

Figure S4. Morphometric analysis of the seminiferous tubules in testicular cryosections. Diameter of the seminiferous tubule (A), and seminiferous tubule cross-sectional area (B). Data are represented as mean ± S.D. (n=6-9 animals per group).
